# Medication Adherence and Influencing Factors Among Patients With Severe Mental Disorders in Low-Income Families During COVID-19 Outbreak

**DOI:** 10.3389/fpsyt.2021.799270

**Published:** 2022-01-18

**Authors:** Huiying Wang, Fengju Yao, Hailing Wang, Changhong Wang, Zhengjun Guo

**Affiliations:** ^1^The Second Affiliated Hospital of Xinxiang Medical University, Henan Mental Hospital, Xinxiang, China; ^2^Henan Key Laboratory of Biological Psychiatry, Xinxiang Medical University, Xinxiang, China

**Keywords:** medication adherence, COVID-19, severe mental disorders, low-income families, regular medication rate

## Abstract

**Background:**

The COVID-19 has spread across the globe in a short time and affected people's life, especially patients with severe mental disorders. Poor adherence to antipsychotics was usually associated with an increasing risk of relapse. This study investigated medication adherence status among patients with severe mental disorders in low-income families during COVID-19 outbreak and the influencing factors.

**Methods:**

To select patients with severe mental disorders in low-income families in central China's Henan Province, we used multi-stage stratified random sampling method. Trained interviewers and psychiatrists collected questionnaire responses from the patients through face-to-face interviews or video interviews. Logistic regression models were used to examine factors that influence the status of medication adherence.

**Results:**

A total of 24,763 valid questionnaires were collected between March 10, 2020, and March 31, 2020. The regular medication rate of patients with severe mental disorders in low-income families during the COVID-19 outbreak was 51.46%. Twelve factors were found to influence medication adherence of investigated individuals. Positive factors for regular medication were younger age, higher education level of patients and their guardians, higher medical expenditure, higher level of self-care ability, having subsidies for care and supervision, having disability certificate and personal care, etc.

**Conclusions:**

The COVID-19 outbreak affected the medication adherence among patients with severe mental disorders in low-income families. The influencing factors are complicated and diverse, including psychological effects, traffic impact, and economy, etc. The government should pay more efforts on social assistance programs and flexibly deal with difficulties during public health emergencies like the COVID-19.

## Introduction

The Corona Virus Disease 2019 (COVID-19) has spread across the globe in a short time and affected people's life (1, 2). From the start of COVID-19 outbreak to early March, most people in China reduced interpersonal interactions and stayed at home, both of which have been found to increase mental health risks, such as increased anxiety and fear (3). For patients with severe mental disorders, such drastic changes could lead to a series of mental symptoms, including depression, unstable mood, and irritability, and even result in fluctuation and deterioration of mental disorders (2).

In China, six diseases are defined as severe mental disorders, including schizophrenia, schizoaffective disorder, bipolar disorder, delusional disorder, intellectual disability with mental disorders and psychotic disorder due to epilepsy (4). According to the latest China Mental Health Survey, there are more than 16 million individuals with severe mental disorders in China (5). These large amount of patients with severe mental disorders leads to a number of significant public health, economic and social problems (6, 7). It is very important to persist long-term and regular treatment for reducing the immense problems and improving the life quality of these patients.

For patients with severe mental disorders in community, the cornerstone of treatment is antipsychotic medication, both in acute and maintenance phases (8). Due to the particularity of antipsychotics, the adherence to treatment is extremely important for the effectiveness of these drugs (8–10). However, it is difficult for many patients with severe mental disorders to take antipsychotic medication as prescribed on a long-term basis (9). Medication non-adherence is a significant problem and one of the most challenging aspects in the maintenance treatment of severe mental disorders (11, 12). Although various antipsychotics are available, non-adherence to these drugs has been shown to be high (12–14). Rate of non-adherence to antipsychotic medication has been reported to range from 8.1 to 62.5% depending on the clinical definition and assessment of adherence, duration of study, and study population, with estimated around 50% non-adherence rate (11, 12, 14, 15). Poor adherence reduces the effectiveness of treatment and is associated with a much higher risk of relapse and lower quality of life, which may induce more public security accidents (8, 11, 15).

Long-term maintenance treatment of severe mental disorders brings a heavy economic burden to patients' families (16, 17). Growing evidence shows that patients with mental illness are more likely to drift into or remain in poverty, forming a negative cycle (18, 19). Because of the huge population of China, a large number of families which have one or more family members with severe mental disorders live in poverty (19). The Chinese government has made a lot of efforts to reduce the heavy economic and social burden of these families and ensure the life quality of patients with severe mental disorders over the last two decades (4, 20). A series of social assistance programs were launched to reinforce community-based healthcare (4, 20). Take the “Central Government Support for the Local Management and Treatment of Severe Mental Illnesses Project” (also known as “686 Project”) as an example. The “686 Project” was established based on the WHO's recommended strategy for integrating hospital-based and community-based mental health services and launched in 2004 following the SARS epidemic (4, 20, 21). The main contents of the “686 Project” are as follows: registration, initial assessment of patients with severe mental disorders, and regular follow-up interviews; free medication and emergency hospitalization; training of relevant personnel (21). Now the project has been applied to the whole country to decrease the societal and economic costs of individuals with severe mental disorders in low-income families and increase the proportion of patients who remain stable (21). However, few studies reported the effects of these social assistance programs on medication adherence of these individuals.

With the occurrence of the COVID-19 pandemic, low-income families which has patients with severe mental disorders has been plunged into extreme difficulties. The traffic restrictions and quarantine measures led to fewer jobs opportunities and less income, as well as less access to mental health services and harder to obtain antipsychotic medicine (2). During the COVID-19 outbreak, low-income families should deal with a number of difficulties, including having fewer or no income, confining patients with severe mental disorders to their homes, and ensuring needed antipsychotics at home. Because of the lockdown, a large number of outpatients couldn't go to hospital and obtain antipsychotics as usual. Some of these patients obtained drugs by express delivery from hospital whereas others couldn't get antipsychotics and stop taking medicine. In addition, if one or more family members got COVID-19, other family members would have tremendous psychological problems, which might have a great negative impact on the life of patients with severe mental disorders and medication adherence (2). Therefore, patients with severe mental disorders in low-income families were facing more difficulties to adhere to the maintenance treatment which may result in relapse and uncontrollable behaviors during the COVID-19 outbreak. However, no data of medication adherence are currently available for the specific population during a pandemic outbreak.

The present study focused on the medication adherence status among patients with severe mental disorders in low-income families during COVID-19 outbreak. In the pandemic outbreak period, irregular medication could induce relapse as well as higher risk of viral infection. The finding of this study will provide evidence for the management of patients with severe mental disorders under major public health events.

## Subjects and Methods

### Subjects

This study recruited patients with severe mental disorders in low-income families in Henan Province, China. The inclusion criteria were: (1) local resident; (2) age ≥ 15 years old; (3) have the ability of autonomous language expression and voluntarily accept this survey; (4) diagnosed with mental disorders according to ICD-10, including schizophrenia, schizoaffective disorder, bipolar disorder, delusional disorder, intellectual disability with mental disorders and psychotic disorder due to epilepsy; (5) patients documented in the Henan Provincial Management and Treatment Office of Severe Mental Disorders; (6) income ≤ ¥3,747 yuan/person/year. The exclusion criteria were: (1) organic mental disease or serious physical disease; (2) loss of follow-up; death of patients. This study was approved by the Ethics Committee of the Second Affiliated Hospital of Xinxiang Medical University, and subjects or their guardians provided their written informed consent.

### Sampling Method

The multi-stage stratified random sampling method was used to select patients with severe mental disorders in low-income families in central China's Henan Province (5). The sampling followed a tiered process, which included four stages. Stage 1, all counties or districts in the 18 cities and 10 province-governing counties of Henan Province were set as the sampling frame. It included a total of 66 districts and 108 counties. Stage 2, patients with severe mental disorders in low-income families were reported to the Henan Provincial Management and Treatment Office of Severe Mental Disorders from village, town, county and city level by level. A random number was assigned to each patient based on their districts and counties. Stage 3, within each district and county, 1/5 of the patients were selected using the random number table. Finally, 25,100 patients with severe mental disorders from low-income families were selected. Stage 4, the management and treatment office of severe mental disorders in each district and county organized trained interviewers and psychiatrists to contact the selected patients and complete the questionnaire through face-to-face or video interviews.

### Questionnaire

According to previous relevant studies (4, 8), practical work experience and the situation of local pandemic prevention and control, the questionnaire was jointly designed by a number of epidemiologists and psychiatrists. Before the beginning of the provincial-wide survey, the questionnaire was evaluated through a small-scale survey. Then the questionnaire was revised to ensure the validity. The filling time of each questionnaire is about 18 min, which is acceptable for patients with severe mental disorders. It consisted of 34 questions in three parts. Part 1, basic information of patients and their families, included age, marital status, medication situation, the number of family members, etc. Part 2, COVID-19 related information, included having COVID-19 patient in the family or not, drug acquisition methods during COVID-19 outbreak, etc. Part 3 included questions about the social assistance programs currently received by patients. The questionnaires were filled by trained interviewers and psychiatrists using the Questionnaire Star, an online survey tool. According to local pandemic prevention and control protocols, either face-to-face or video interviews were adopted to collect patient information. For face-to-face interviews, the interviewers must wear masks and keep a distance over 1 meter from the respondents. The medication adherence status within 1 month was determined mainly by the self-report of patients. To ensure the accuracy of the survey, the adherence status of self-report was confirmed with psychiatrist's prescriptions. If the prescriptions were not available, the adherence status of self-report was confirmed with their guardians. If patients took medicine according to doctor's advice, they were considered as regular medication. If patients took medicine intermittently or refused to take medicine, they were considered as irregular medication.

### Quality Control

The management and treatment offices of severe mental disorders in each city and district/county had either full-time or part-time quality control personnel. The quality control personnel were in charge of summarizing the completion of the questionnaires every day, checking whether there were any omissions, verifying the accuracy and integrity, and reporting the collected information to the Henan Provincial Management and Treatment Office of Severe Mental Disorders. All of the questionnaires were audited on the same day as they were collected. If the interviewers cannot get any responses from the patients or their guardians after contacting them three times, the patients were excluded from this data collection.

### Statistical Analysis

Before data analysis, trained statisticians checked the data. All the statistical analyses were conducted using SPSS 26.0. Chi-square tests were used to analyze categorical data. Logistic regression was used to analyze the factors that may influence the medication adherence.

## Results

### Participant Demographics

From March 10, 2020, to March 31, 2020, responses from 24763 patients (response rate: 98.66%) were collected by 2,300 qualified interviewers through face-to-face or online interviews. Table 1 presented the demographics of these patients. Among them, 13,235 (53.45%) were men and 11,528 (46.55%) were women. Most of the patients were in the age group of 41–60 years old (*n* = 12,518, 50.55%), married (*n* = 11,949, 48.25%), diagnosed with schizophrenia (*n* = 19,976, 80.67%), and had the diagnosed mental illness for more than 10 years (*n* = 17,845, 72.06%). Additionally, 207 (0.84%) patients reported having COVID-19 and 379 (1.53%) reported their family member(s) had COVID-19.

**Table 1:** Participant demographics (*n* = 24,763).

	**Frequency, n (%)**
**Gender**	
Male	13,235 (53.45%)
Female	11,528 (46.55%)
**Age (years)**	
≤18	448 (1.81%)
19–40	7,140 (28.83%)
41–60	12,518 (50.55%)
≥61	4,657 (18.81%)
**Marital status**	
Single	9,021 (36.43%)
Married	11,949 (48.25%)
Divorced	2,052 (8.29%)
Widowed	1,741 (7.03%)
**Disease**	
Schizophrenia	19,976 (80.67%)
Schizoaffective disorder	527 (2.13%)
Bipolar disorder	976 (3.94%)
Delusional disorder	197 (0.80%)
Intellectual disability with mental disorders	1,769 (7.14%)
Psychotic disorders due to epilepsy	1,318 (5.32%)
**Duration of disease (years)**
<3	784 (3.17%)
3–5	1,979 (7.99%)
5–10	4,155 (16.78)
≥10	17,845 (72.06%)
**Patient got COVID-19**	
No	24,556 (99.16%)
Yes	207 (0.84%)
**COVID-19 patient in the family**	
No	24,384 (98.47%)
Yes	379 (1.53%)

### Univariate Analysis of Medication Adherence

Of all these patients, 12,743 (51.46%) took medication regularly and 12,020 (48.54%) didn't. Univariate analysis results showed the factors that are different between the medication adherence and non-adherence groups among patients with severe mental disorders in low-income families during COVID-19 outbreak (Table 2). Some factors were found to be different between the two groups include age (χ^2^ = 295.519, *p* < 0.001), marital status (χ^2^ =153.900, *p* < 0.001), disease (χ^2^ = 916.306, *p* < 0.001), duration of disease (χ^2^ = 74.490, *p* < 0.001), education level of patient (χ^2^ = 1,374.867, *p* < 0.001), education level of patient's guardian (χ^2^ = 91.361, *p* < 0.001), personal care (χ^2^ = 307.870, *p* < 0.001), medical expenditure (χ^2^ = 1,031.562, *p* < 0.001), and level of self-care ability (χ^2^ = 216.691, *p* < 0.001).

**Table 2:** Univariate analysis of the medication adherence of patients with severe mental disorders in low-income families (*n* = 24,763).

	**Irregular medication (case)**	**Regular medication (case)**	***χ^2^***	***p***
**Gender**				
Male	6,436	6,799	0.089	0.765
Female	5,584	5,944		
**Age (years)**				
≤18	256	192	295.519	<0.001
19–40	3,014	4,126		
41–60	6,048	6,470		
≥61	2,702	1,955		
**Marital status**				
Single	4,704	4,317	153.900	<0.001
Married	5,495	6,454		
Divorced	852	1,200		
Widowed	969	772		
**Disease**				
Schizophrenia	9,185	10,791	916.306	<0.001
Schizoaffective disorder	292	235		
Bipolar disorder	527	449		
Delusional disorder, psychotic disorder due to epilepsy	96	101		
Intellectual disability with mental disorders	1,440	329		
Psychotic disorder due to epilepsy	480	838		
**Duration of disease (years)**				
<3	423	361	74.490	<0.001
3–5	955	1,024		
5–10	1,776	2,379		
≥10	8,866	8,979		
**Education level of patient (grade)**				
0	6,262	3,828	1,374.867	<0.001
1–6	3,222	4,143		
7–9	2,105	3,957		
10–12	393	694		
Diploma and above	38	121		
**Education level of patient's guardian (grade)**				
<1	4,533	2,911	91.361	<0.001
1–6	4,369	4,838		
7–9	2,724	4,361		
10–12	355	560		
Diploma and above	39	73		
**Medical expenditure (yuan/year)**				
<3,000	8,575	6,356	1,031.562	<0.001
3,000–5,000	2,333	3,888		
5,000–10,000	688	1,576		
≥10,000	424	923		
**Personal care**				
No	4,927	3,863	307.870	<0.001
Yes	7,093	8,880		
**Level of self-care ability**				
No self-care ability	880	621	216.691	<0.001
Few self-care ability	1,526	1,202		
Partial self-care ability	2,906	2,724		
Fully self-care ability	6,708	8,196		

*Regular medication, taking medicine according to doctor's advice; Irregular medication, taking medicine intermittently or refusing to take medicine*.

The social assistance programs available to patients with severe mental disorders in low-income families were analyzed and showed in Table 3. The results suggested that almost all of social assistance grams associated with more patients reporting taking medication regularly, including Subsidies for Care and Supervision of Patients with Severe Mental Disorders (χ^2^ = 27.657, *p* < 0.001), Care and Assistance Leading Group and Case Management Service Team for Patients with Severe Mental Disorders (χ^2^ = 12.352, *p* < 0.001), Family Physician Contract Services (χ^2^ = 6.252, *p* = 0.012), Community Rehabilitation Services (χ^2^ = 56.789, *p* < 0.001), 686 Project(χ^2^ = 90.130, *p* < 0.001), Subsistence Allowances (χ^2^ = 4.716, *p* = 0.030), and other subsidies (χ^2^ = 30.529, *p* < 0.001).

**Table 3:** Impact of social assistance programs on the medication adherence of patients with severe mental disorders in low-income families (*n* = 24,763).

	**Yes/No**	**Irregular medication (case)**	**Regular medication (case)**	***χ^2^***	***p***
Subsidies for care and supervision of patients with severe mental disorders	No	10,640	10,997	27.657	<0.001
	Yes	1,380	1,746		
Care and assistance leading group and case management service team for patients with severe mental disorders	No	8,216	8,443	12.352	<0.001
	Yes	3,804	4,300		
Family physician contract services	No	1,598	1,559	6.252	0.012
	Yes	10,422	11,184		
Community rehabilitation services	No	10,081	10,218	56.789	<0.001
	Yes	19,39	2,525		
686 Project	No	11,341	11,624	90.130	<0.001
	Yes	679	1,119		
Subsistence allowances	No	3,715	4,102	4.716	0.030
	Yes	8,305	8,641		
Other subsidies	No	6,178	6,102	30.529	<0.001
	Yes	5,842	6,641		

*Regular medication, taking medicine according to doctor's advice; Irregular medication, taking medicine intermittently or refusing to take medicine*.

Table 4 showed the impact of COVID-19 outbreak on the medication adherence of patients with severe mental disorders in low-income families. Having COVID-19 patient in the family (χ^2^ = 22.667, *p* < 0.001), patient got COVID-19 (χ^2^ = 5.296, *p* = 0.021), confined patients to their homes (χ^2^ = 187.535, *p* < 0.001), and drug acquisition methods during COVID-19 outbreak (χ^2^ = 12,897.859, *p* < 0.001) were variables significantly different between medication adherence and non-adherence patients.

**Table 4:** Impact of COVID-19 outbreak on the medication adherence of patients with severe mental disorders in low-income families (*n* = 24,763).

	**Irregular medication (case)**	**Regular medication (case)**	***χ^2^*** **value**	***P***
**COVID-19 patient in the family**				
No	11,882	12,502	22.667	<0.001
Yes	138	241		
**Patient got COVID-19**				
No	11,936	12,620	5.296	0.021
Yes	84	123		
**Confined patients to their homes during COVID-19 outbreak**				
No	2,248	1,581	187.535	<0.001
Yes	9,772	11,162		
**Drug acquisition methods during COVID-19 outbreak**				
No medication	8,157	0	12,897.859	<0.001
Hospital	2,327	7,886		
Express delivery	1,536	4,857		

*Regular medication, taking medicine according to doctor's advice; Irregular medication, taking medicine intermittently or refusing to take medicine*.

### Logistic Regression Analysis for Medication Adherence

Logistic regression analysis was used to analyze the influencing factors of medicine adherence among patients with severe mental disorders in low-income families during COVID-19 outbreak. The dependent variable was medication adherence (no = 0, yes = 1). The independent variables included 22 factors that are significantly different between the two medication-adherence groups as suggested by the univariate analysis reported above. The overall model was evaluated by two inferential statistical tests: the Omnibus test (χ^2^ = 16,966.716, *p* < 0.001) and Wald test (χ^2^ = 21.103, *p* < 0.001). They yielded similar conclusions suggesting that the logistic regression model was effective. The Hosmer and Lemeshow test was presented in Table 5. The inferential goodness-of-fit test yielded a χ^2^ of 3.831 and was insignificant (P = 0.872), suggested that the logistic regression model in this study was fit well to the data.

**Table 5:** Logistic regression analysis of the factors that may influence the medication adherence of patients with severe mental disorders in low-income families (*n* = 24,763).

	***B***	***SE***	***Wald χ^2^***	***p***	***OR***
**Age (years)**					
≥61 (reference group)			26.957	<0.001	
≤18	0.415	0.171	5.901	0.015	1.514
19–40	0.308	0.061	25.220	<0.001	1.360
41–60	0.164	0.053	9.590	0.002	1.178
**Disease**					
Psychotic disorder due to epilepsy			38.655	<0.001	
Schizophrenia	−0.234	0.083	7.999	0.005	0.792
Schizoaffective disorder	−0.710	0.142	25.03	<0.001	0.492
Bipolar disorder	−0.429	0.123	12.212	<0.001	0.651
Delusional disorder	−0.477	0.210	5.149	0.023	0.621
Intellectual disability with mental disorders	−0.523	0.127	16.917	<0.001	0.593
**Duration of disease**					
≥10			8.749	0.033	
<3	−0.135	0.108	1.577	0.209	0.874
3–5	−0.186	0.069	7.393	0.007	0.830
5–10	−0.060	0.051	1.348	0.246	0.942
**Medical expenditure (yuan/year)**					
<3,000			129.984	<0.001	
3,000–5,000	−0.575	0.085	46.081	<0.001	0.563
5,000-10,000	−0.218	0.088	6.109	0.013	0.804
≥10,000	−0.024	0.100	0.059	0.807	0.976
**Subsidies for care and supervision**	0.159	0.056	8.110	0.004	1.172
**Education level of patient (grade)**					
Diploma and above			30.873	<0.001	
<1	−0.685	0.277	6.119	0.013	0.504
1-6	−0.538	0.276	3.812	0.051	0.584
7-9	−0.379	0.276	1.895	0.169	0.684
10-12	−0.311	0.287	1.172	0.279	0.733
**Education level of patient's guardian (grade)**					
Diploma and above			20.890	<0.001	
<1	−0.299	0.319	0.877	0.349	0.742
1-6	−0.199	0.318	0.393	0.531	0.819
7-9	−0.042	0.318	0.017	0.896	0.959
10-12	−0.316	0.329	0.921	0.337	0.729
**Level of self-care ability**					
Fully self-care ability			110.063	<0.001	
No self-care ability	−0.525	0.084	38.742	<0.001	0.592
Few self-care ability	−0.501	0.065	59.753	<0.001	0.606
Partial self-care ability	−0.399	0.048	69.074	<0.001	0.671
**Disability certificate**	−0.151	0.045	11.153	0.001	0.860
**Personal care**	−0.321	0.042	59.317	<0.001	0.726
**COVID-19 patients in the family**	−0.488	0.246	3.931	0.047	0.614
**Confined patients to their homes during COVID-19 outbreak**	−0.341	0.052	43.551	<0.001	0.711
**Overall model evaluation**		***χ^2^***	**d**f	***P***	
Omnibus test		16966.716	46	<0.001	
Wald test		21.1031	<0.001		
**Goodness-of-fit test**					
Hosmer & Lemeshow		3.831	8	0.872	

It showed that medicine adherence among patients with severe mental disorders in low-income families during COVID-19 outbreak was significantly affected by 12 factors, including having COVID-19 patient in the family, confined patients to their homes during COVID-19 outbreak, duration of mental disorders, level of self-care ability, education level of patient, etc. The results were shown in Table 5.

The validity of logistic regression model was examined by comparing the observed and predicted medication adherence status. As shown in Table 6, the sensitivity was 99.68% and the specificity was 68.34%. Meanwhile, the false positive rate was 23.05% and the false negative rate was 0.50%. Overall, the percentage of correct prediction was 84.47%, which is a much higher than the chance level.

**Table 6:** The observed and the predicted^*^ medication adherence of patients with severe mental disorders in low-income families (*n* = 24,763).

**Observed**	**Predicted**		**% Correct**
	**Regular medication**	**Irregular medication**	
Regular medication	12,702	41	99.68
Irregular medication	3,805	8,215	68.34
Overall % correct			84.47
	Sensitivity = 99.68%. Specificity = 68.34%.		
	False positive = 23.05%. False negative = 0.50%.		

* *The predicted medication adherence status is assigned using the cutoff of 0.50, i.e., predicted regular medication = logistic regression predicted probabilities > 0.50, and predicted irregular medication = predicted probabilities < 0.50*.

## Discussion

This study investigated the medication adherence and influencing factors among patients with severe mental disorders in low-income families in the Central China's Henan Province during the epidemic outbreak. We found that 51.46% of the patients had regular adherence, while 48.54% of the patients didn't take antipsychotics as prescribed. Twelve factors were found to influence medication adherence of investigated individuals significantly. Positive factors for regular medication were younger age, higher education level of patients and their guardians, higher medical expenditure, higher level of self-care ability, having subsidies for care and supervision, having disability certificate and personal care, etc.

Univariate analysis showed that almost all of the social assistance programs significantly associated with higher medication adherence rate. The finding was similar to a recent study (8). Previous studies also showed that incentives, especially financial incentives, improved the adherence to maintenance treatment in patients with psychotic disorders (9, 22). There were several possible explanations. First, the social assistance programs partly solved the patients' economic problems at home. Second, some relief policies encouraged patients to keep taking medicines regularly. For example, the “Subsidies for Care and Supervision of Patients with Severe Mental Disorders” require the patient's guardians to sign an agreement with the government. The agreement clearly stipulated that the guardians must take care of the patient daily, supervise the patient to take medicine regularly, and monitor the patient's condition at any time. In addition, the study found that social assistance programs only covered a small part of the patients and had different efficiency for improving regular adherence. The “686 Project” was showed to have the highest adherence rate in our study. Therefore, it would be helpful to enhance the medication adherence by expanding assistance efforts and intensifying inclination of policy for social assistance programs which have better efficiency to help more patients with severe mental disorders in low-income families during the pandemic.

For the patients with severe mental disorders in low-income families, besides poverty, personal care was another main factor affecting the patient's basic life. Compared with patients who have someone to take care of their daily life, patients with no personal care had a lower regular medication rate. Even with supporting policies, it is hard for the patients with no personal care to take medicine regularly in the long-term (23). Personal care could look after patients, care for their conditions, and supervise their medication. Our findings suggested that the management should be strengthened for the patients who have weak guardianship, and more management methods, such as building a guardianship network (24), could be explored.

This study found that guardians' and patients' education levels influenced patients' medication adherence. Lower education levels were risk factors, and the patients with higher level of education background showed better medication adherence. One possible reason is that highly educated guardians and patients better understand the diseases and realize the importance of regular medication (8). In addition, highly educated guardians and patients tend to follow doctors' advice and have better doctor-patient relationships (25).

This study also showed that age was one of the factors affecting regular medication. Compare to patients older than 60 years, the regular medication rate was higher in the younger patients, consistent with previous studies (11, 15). Reasons could be as follows: First, younger patients have less knowledge of the medication and may need more supervision to take drugs regularly. Second, with increasing age, patients may realize the necessity of treatment and maintain regular medication. Third, most of the older patients having their spouses as their guardians, who often have limited guardianship capacity. Some older patients live alone, and no one supervises them to take medicine regularly.

Additionally, we found that lack of self-care ability was another risk factor for medication adherence. The regular medication rate gradually decreased with increasing self-care ability. Patients with a higher level of self-care ability were willing to participate in more self-care activities, which increased their feelings of well-being and medication adherence (16).

In particular, the study investigated the impact of COVID-19 outbreak on the regular medication of patients with severe mental disorders in low-income families. The regression analysis showed that whether there were COVID-19 patients in the family had little effect on medication adherence. One possible explanation is that there were not many individuals infected by COVID-19 in Henan Province, and these people received timely and proper treatment. Furthermore, the drug acquisition methods during COVID-19 outbreak were investigated in the present study. The method for drug acquisition, express delivery, was shown to have similar effect on medication adherence. It is suggested that express delivery is a good replacement method of going to hospital as usual and it can also be used during the pandemic. The government could expand the publicity of the method and let more patients and their guardians know how to obtain antipsychotics during lockdown.

In conclusion, regular medication of patients with severe mental disorders in low-income families was affected during the outbreak of COVID-19. The influencing factors are complicated and diverse, including psychological effects, traffic impact, and economy, etc. Besides meeting the fundamental demands of patients in low-income families, the government should pay attention to the psychological problems of these patients and their guardians and develop suitable drug acquisition methods during public health emergencies like the COVID-19.

This study had some limitations. First, the sampling of this study focused on the patients documented in the Henan Provincial Management and Treatment Office of Severe Mental Disorders, whereas other patients were not included. The sample included in this study may not be representative of the whole population. Second, the status of medication adherence in the study were recorded according to the answers of patients or their guardians without objective evaluation. Third, this study was a cross-sectional trial during the outbreak of COVID-19. More studies should be done in future.

## Data Availability Statement

The raw data supporting the conclusions of this article will be made available by the authors, without undue reservation.

## Ethics Statement

The studies involving human participants were reviewed and approved by Ethics Committee of the Second Affiliated Hospital of Xinxiang Medical University. Written informed consent to participate in this study was provided by the participants' legal guardian/next of kin.

## Author Contributions

ZG and CW designed the study and wrote the study protocol. ZG, HuW, FY, and HaW acquired the data and performed the statistical analyses. HuW and ZG interpreted the data and drafted the initial manuscript. All authors contributed in the final drafting and critically revised the manuscript.

## Funding

This study was funded by Henan Medical Science and Technology Research Plan Joint Construction Project (LHGJ20200531 and 2018020375) and Open Program of Henan Key Laboratory of Biological Psychiatry (ZDSYS2019009).

## Conflict of Interest

The authors declare that the research was conducted in the absence of any commercial or financial relationships that could be construed as a potential conflict of interest.

## Publisher's Note

All claims expressed in this article are solely those of the authors and do not necessarily represent those of their affiliated organizations, or those of the publisher, the editors and the reviewers. Any product that may be evaluated in this article, or claim that may be made by its manufacturer, is not guaranteed or endorsed by the publisher.
